# AI-based methods for the assessment of DNA damage and repair mechanisms

**DOI:** 10.3389/fsysb.2026.1734322

**Published:** 2026-04-29

**Authors:** Paola Lecca, Michela Lecca, Adaoha Elizabeth Ihekwaba-Ndibe

**Affiliations:** 1 Faculty of Engineering, Free University of Bozen-Bolzano, Bolzano-Bozen, Italy; 2 Technologies of Vision Unit at Digital Industry Center, Fondazione Bruno Kessler, Trento, Italy; 3 School of Sciences, Coventry University, Coventry, United Kingdom

**Keywords:** Bayesian artificial neural networks, comet assay, deterministic artificial neural networks, DNA repair, recurrent neural networks

## Abstract

In recent years, a growing number of artificial intelligence (AI)–driven approaches have been developed to elucidate chemico-biological interactions associated with DNA damage and oxidative stress. Deep learning–based techniques, in particular, have demonstrated substantial potential within molecular biology and toxicology. As a result, researchers and clinicians alike hold high expectations that AI-enabled tools will soon make meaningful contributions to our understanding of the molecular and cellular mechanisms governing DNA damage and repair. In this article, we present a concise yet comprehensive overview of the computational methodologies underpinning contemporary deep learning approaches. We examine their capacity to support DNA damage assessment by revealing mechanistic insights into damage induction and response pathways. Particular emphasis is placed on deep learning techniques designed to enhance the analysis of complex biological data, including the automated detection and quantification of DNA damage from *comet assay* images and microscopy-based platforms. Furthermore, we critically assess the extent to which a gap exists between the expectations of researchers, biologists, and clinicians and the current practical capabilities of AI technologies in this domain. Finally, we offer a forward-looking perspective on how this gap might be narrowed, outlining key methodological, data-driven, and translational challenges that must be addressed to fully realize the potential of AI in DNA damage and repair research.

## 1 Introduction

Computational challenges in DNA repair bioinformatics involve managing the vast volume and complexity of data generated by DNA repair studies, integrating diverse biological data types—including genomics, proteomics, transcriptomics, and other omics modalities—to elucidate repair mechanisms, and developing algorithms specifically tailored to simulate complex DNA repair processes and predict the consequences of DNA damage or repair deficiencies. Although challenges related to the integration of data from different sources, experimental platforms, and data modalities are highly topical in the study of DNA repair mechanisms, this forward-looking article places greater emphasis on issues associated with the detection and assessment of DNA damage through computational modeling approaches. The primary goals of these approaches are the description, explanation, and prediction of the agents and processes that govern the delicate and essential mechanisms responsible for maintaining genome integrity. Data integration—often a preparatory step for modeling and computational simulation of DNA repair interactions—has been extensively addressed in the literature and is discussed in numerous existing studies (Tummler and Klipp, 2024; Stanic and Mekhail, 2022; Sherill-Rofe et al., 2022; Hall and Niarakis, 2021; Liu et al., 2016; Dolan et al., 2015).

**FIGURE 1:**
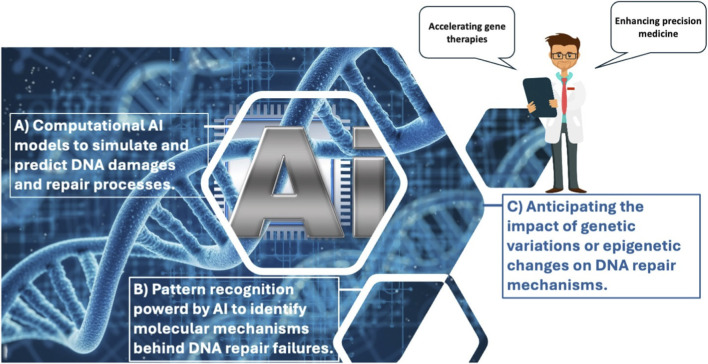
Artificial intelligence (AI)-based computational and statistical methods are increasingly important for identifying and understanding the mechanisms underlying DNA damage and repair [objectives **(A,B)**]. These capabilities support the achievement of the clinical objective **(C)**, which focuses on anticipating the impact of genetic variations and epigenetic changes on DNA repair mechanisms. Progress toward this objective has the potential to accelerate the development of gene therapies and advance precision medicine.

For completeness, we briefly mention several AI techniques commonly used for integrating heterogeneous biological datasets, which are increasingly applied in studies of DNA damage and repair mechanisms. Machine learning methods, for example, can automate schema mapping, identify patterns across datasets, and detect anomalies in complex data streams. As discussed by (Sartori et al., 2025; Sibilio et al., 2025), supervised learning approaches can correlate domains using previously annotated data, whereas unsupervised learning methods group similar records to facilitate entity resolution across heterogeneous databases. Natural language processing (NLP) techniques further enable the extraction of biologically relevant relationships from unstructured text sources, such as scientific literature and experimental reports; (Kumar and Mukhtar, 2025), for instance, describe an NLP-based method for mining gene–function relationships from published articles. Deep learning algorithms can also analyse large and complex datasets to uncover patterns overlooked by conventional methods, and are frequently used for tasks such as data deduplication and entity matching (Ballard et al., 2024). Finally, knowledge graphs and semantic models provide a framework for linking entities from diverse biological systems—such as genes, proteins, and functional complexes—into unified representations that enhance contextual interpretation of biological data (Kratz et al., 2023; Hu et al., 2026). Beyond facilitating technical integration of heterogeneous datasets, such AI-based approaches also support the generation of mechanistic hypotheses by revealing hidden relationships across DNA damage response pathways and repair mechanisms. Methods based on artificial intelligence (AI), including machine learning and deep learning, are transforming the detection and assessment of DNA damage and repair by streamlining the analysis of complex experimental data derived from techniques such as immunofluorescence microscopy and single-cell gel electrophoresis, commonly known as the *comet assay* introduced in Section 5. The *comet assay* is a widely used method in molecular and cellular biology for detecting DNA damage (Olive and Banáth, 2006). AI-based approaches analyze imaging and molecular datasets to identify and quantify DNA lesions, predict chemico-biological interactions, and model DNA repair kinetics, thereby improving analytical accuracy and enabling high-throughput assessment. The evaluation of DNA damage—and, where feasible, the prediction of damage outcomes—using AI-driven computational techniques becomes possible when the following objectives are met, the first two of which support the achievement of the third (see Figure 1)). Achieving these objectives enables AI not only to improve the accuracy and efficiency of DNA damage detection and analysis but also to support the development of predictive and mechanistic models of DNA repair processes. In this way, AI-based approaches can transform traditionally slow, error-prone, or manual molecular biology workflows into integrated systems capable of automated analysis, hypothesis generation, and predictive modeling.

- The development of advanced computational models and algorithms is essential for simulating DNA damage and repair processes, predicting the effects of mutations in repair genes, and understanding interactions among different repair pathways. Such models are critical for moving from descriptive to mechanistic and predictive understanding. Recent studies (2020–2024) in computational toxicology and systems biology have employed neural network–based simulations to analyze the dynamics of double-strand break repair (DSBR) and base excision repair (BER). We refer the reader to (Walter et al., 2024; Kleinstreuer and Hartung, 2024; Karimi Zeverdegani et al., 2024; Pantic et al., 2023; Jia et al., 2023; Sharma et al., 2023; Tetko et al., 2022; Green et al., 2021; Hemmerich and Ecker, 2020) for a comprehensive overview. Of particular interest for their innovative contribution to the bioinformatics landscape are approaches that combine deep learning with agent-based modeling. These hybrid frameworks enable the simulation of multistep DNA repair processes—such as damage recognition, incision, re-synthesis, and ligation—while capturing their dynamic and stochastic behavior (Sivakumar et al., 2022; Gu et al., 2021; Cogno et al., 2024; Stephan et al., 2024).

Agent-based modeling has already been applied to simulate DNA damage response dynamics and treatment responses in cancer systems, including models of ATR-inhibitor therapies and tumor growth under DNA damage stress (Hamis et al., 2021). More recently, agent-based frameworks have been combined with molecular and gene-expression data to improve predictive modeling of disease progression and therapeutic outcomes (Sridharan and Ghosh, 2025). Despite their potential, integrating deep neural networks with agent-based simulations presents important methodological challenges. Neural models typically learn statistical relationships from large datasets, whereas agent-based models require explicitly defined interaction rules and parameters describing the behavior and interaction of individual agents. Translating learned representations into biologically meaningful agent behaviors therefore remains computationally demanding and often requires careful calibration and validation against experimental data. Recent surveys on AI agents in biological research highlight both the opportunities and the technical challenges associated with these hybrid modeling paradigms (Qi et al., 2026). In parallel, emerging work on AI-driven genotoxicity assessment illustrates how integrating mechanistic simulations with machine-learning approaches may enhance predictive modeling of DNA damage responses and toxicological outcomes (Barghi et al., 2026).

B. Identifying the molecular mechanisms underlying DNA repair failure in disease contexts—including cancer and inherited DNA repair deficiency syndromes—requires robust bioinformatic workflows. Numerous cancers and rare disorders, such as xeroderma pigmentosum, ataxia telangiectasia, and Fanconi anemia, arise from defects in DNA repair pathways. Multi-omics integration, pathway analysis, and protein–protein interaction network modeling are essential for identifying where and how these failures occur. AI-based pattern recognition methods can further uncover latent molecular signatures linking specific repair deficiencies to distinct clinical phenotypes (Alum, 2025; Fountzilas et al., 2025; Mak et al., 2025; Sciaccotta et al., 2025).
C. Anticipating the impact of genetic variation and epigenetic modification on DNA repair mechanisms requires sophisticated statistical and machine learning approaches. DNA repair pathways are tightly regulated by genetic variants (e.g., polymorphisms and mutations) and epigenetic factors (e.g., DNA methylation and histone modifications). Statistical modeling and machine learning techniques—such as regression models, random forests, and Bayesian networks—are increasingly used to predict the functional consequences of variants in DNA repair genes and their pathogenic relevance, including genes such as *ATM* (Lee et al., 2025), *BRCA1* (Li et al., 2023; Kang et al., 2023; Hart et al., 2020), and *XRCC1* (Choudhary et al., 2023).

Taken together, these objectives outline a unified computational framework in which AI-based methods enable the integration of heterogeneous biological data with predictive and mechanistic models of DNA repair dynamics.

AI techniques for evaluating DNA damage are attracting increasing attention due to their broad applicability in areas such as drug development, cancer detection, and chemical safety assessment. The integration of AI into both research and clinical practice related to DNA damage and repair is becoming increasingly common, particularly in automated image analysis, precision diagnostics, and genotoxicity evaluation. Machine learning approaches are beginning to complement traditional bioassays by predicting how novel agents—including pharmaceuticals and engineered nanoparticles—may induce DNA damage, thereby reducing reliance on animal testing while improving scalability and predictive performance.

Although foundational applications of AI to DNA damage and repair emerged between 2018 and 2020, recent developments indicate a shift toward deeper mechanistic insight. A notable example is the Pythia model (Naert et al., 2025), which demonstrated that DNA repair outcomes following CRISPR/Cas9-induced double-strand breaks are strongly influenced by local DNA sequence context rather than being purely stochastic. By learning sequence-dependent insertion and deletion patterns, Pythia revealed biases toward microhomology-mediated end joining at specific loci. These predictions were experimentally validated using high-throughput sequencing, confirming concordance between model predictions and observed repair outcomes.

In parallel, large language model–based tools such as CRISPR-GPT (Qu et al., 2025) have emerged as integrative research assistants rather than direct mechanism-discovery engines. CRISPR-GPT supports genome-editing design and optimization by synthesizing existing experimental knowledge, analyzing complex DNA repair–related datasets, and assisting in the interpretation of repair outcomes during gene therapy development. While such tools do not directly uncover new DNA repair mechanisms, they significantly enhance hypothesis generation, experimental planning, and decision-making within established repair frameworks. Similarly, AI-HOPE-TP53 (Yang et al., 2025) enables pathway-focused exploration of TP53-associated molecular alterations in colorectal cancer by integrating curated genomic and clinical datasets with natural language–based queries, accelerating biological interpretation in precision oncology.

These examples, among many others, illustrate the diverse contexts in which AI can support DNA damage assessment, pathogenicity determination, and the study of repair mechanisms.

The rapid evolution and increasing integration of AI into DNA damage research make it essential to inform clinicians, biologists, and researchers of both the current state of the art and near-term prospects—constituting the primary motivation for this review. This article is grounded in the observation that most mathematical and computational AI methods are inherently adaptable to a wide range of DNA damage–related scenarios and data types. These approaches are sufficiently general to be applied across multiple repair mechanisms, including direct damage reversal (e.g., via O6-methylguanine methyltransferase), base excision repair, and nucleotide excision repair. However, such flexibility does not imply uniform applicability without modification. To extract biologically meaningful insight and achieve high predictive accuracy, each method must be appropriately tailored to the specific biological question and dataset. Accordingly, this review focuses primarily on deep neural networks, which represent one of the most versatile computational frameworks underpinning modern AI. We provide a mathematical overview of these models, discuss their principal applications in DNA damage and repair research, and highlight the emerging challenges they must address. In particular, we examine how classical mathematical frameworks—such as Bayesian statistics—can be integrated into artificial neural networks to enhance inference, enabling more reliable estimation of damage severity and repair dynamics even in the presence of stochasticity and uncertainty. Finally, we devote a dedicated section to deep learning–based image analysis for DNA damage detection, illustrating how AI can automate and standardize interpretation of *comet assays* and fluorescence microscopy, which are otherwise time-consuming and susceptible to operator-dependent variability.

## 2 Overview of DNA damage and repair mechanisms

### 2.1 Biological overview of DNA damage and repair pathways

DNA integrity is continuously challenged by endogenous processes such as DNA replication errors, oxidative metabolism, and spontaneous base hydrolysis, as well as by exogenous agents including ultraviolet (UV) radiation, ionizing radiation, and chemical mutagens (Jackson and Bartek, 2009). To preserve genomic stability, cells rely on an integrated DNA damage response (DDR) that detects DNA lesions, coordinates cell-cycle checkpoints, and activates appropriate repair pathways through extensive signaling networks (Bryant et al., 2005; Matthews et al., 2021). Central to this response are damage sensors and transducers such as ataxia telangiectasia mutated (ATM), ATM and Rad3-related (ATR), and poly(ADP-ribose) polymerase 1 (PARP1), which orchestrate downstream repair and signaling events (Bryant et al., 2005; Ciccia and Elledge, 2010; Farmer et al., 2005; Groelly et al., 2022; Jackson and Bartek, 2009). Multiple DNA repair pathways have evolved to resolve distinct classes of DNA lesions (Lam, 2022; Lord and Ashworth, 2012; Tong et al., 2024). Although the core enzymatic steps of these pathways are well characterized, their regulation, coordination, and pathway choice remain incompletely understood. Importantly, DNA repair pathways do not operate in isolation. Extensive crosstalk exists between repair mechanisms, chromatin remodeling, transcriptional regulation, and cell-cycle control (Matthews et al., 2021). Repair outcomes are further influenced by stochastic effects, spatial organization within the nucleus, and dynamic changes in chromatin structure (Mohan et al., 2021; Uphoff et al., 2016; Di Stefano and Cavalli, 2022; Sanders et al., 2020). These features pose significant challenges for quantitative modeling and limit the extent to which mechanistic insight can be derived from reductionist experimental approaches alone. Consequently, there is growing interest in computational and AI-based methods capable of integrating heterogeneous biological data to infer pathway activity, repair outcomes, and damage severity across diverse conditions.

**FIGURE 2:**
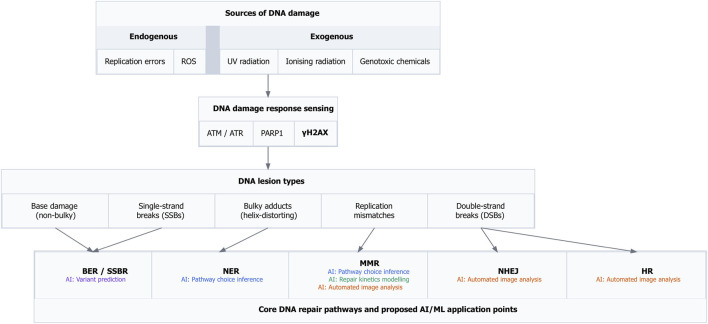
DNA damage types, repair pathways, and proposed AI/ML application points. DNA damage arises from endogenous sources, including replication errors and reactive oxygen species (ROS), and from exogenous agents such as ultraviolet radiation, ionising radiation, and genotoxic chemicals. These insults generate lesions including base damage, single-strand breaks (SSBs), bulky adducts, replication mismatches, and double-strand breaks (DSBs). Damage sensing involves key DNA damage response (DDR) regulators such as ATM/ATR, PARP1, and γ H2AX. Lesions are repaired through base excision repair and single-strand break repair (BER/SSBR), nucleotide excision repair (NER), mismatch repair (MMR), non-homologous end joining (NHEJ), and homologous recombination (HR). The figure highlights proposed points where artificial intelligence (AI) and machine learning (ML) approaches may support variant pathogenicity prediction, pathway choice inference, repair kinetics modelling, and automated image-based analysis.

Damage sensing and signaling are coordinated by central DDR regulators such as ATM, ATR, and PARP1, which integrate cell-cycle status, chromatin context, and lesion complexity to influence repair pathway choice and repair outcomes (Huber et al., 2004; Ciccia and Elledge, 2010; Marechal and Zou, 2013). Figure 2 highlights key stages at which AI and machine-learning approaches are commonly applied, including variant pathogenicity prediction, inference of repair pathway engagement, modeling of repair kinetics, and automated quantification of DNA damage from imaging data (e.g., *comet assays* and fluorescence microscopy). Together, these applications underscore the biological complexity and regulatory crosstalk that motivate the use of AI-based computational methods discussed in this review.

### 2.2 Statistical and machine learning methods

Statistical and classical machine-learning approaches have long formed the foundation of computational analyses of DNA damage and repair (Alexandrov et al., 2020; Chen et al., 2019; Davies et al., 2017; Liu et al., 2022; Mourad et al., 2018). These models remain attractive due to their robustness in modest sample-size settings, interpretability of covariates, and compatibility with established validation frameworks. Widely used techniques include Bayesian networks (BNs), logistic regression, decision trees, random forests, and support vector machines (SVMs), each offering distinct advantages for applications in toxicogenomics, variant classification, and oxidative stress modeling.

Bayesian networks capture conditional dependencies among biological variables, enabling probabilistic inference of unobserved processes such as pathway activation from transcriptomic or epigenomic data (Fernandez et al., 2025; Gogoshin et al., 2021; Jennen et al., 2015). In DNA repair research, BNs can represent causal relationships among DNA damage sensors (ATM/ATR), transducers (CHK1/CHK2, PARP), and downstream effectors, supporting probabilistic reasoning about pathway engagement and biomarker prioritization (Isci et al., 2014). Regularized logistic regression models, including L1/L2 and elastic-net variants, continue to perform strongly in binary classification tasks (Kang et al., 2023; Torang et al., 2019) and provide interpretable effect estimates suitable for risk stratification.

Decision-tree algorithms offer intuitive, rule-based insights (Jiang et al., 2024) but are prone to overfitting in small datasets. Random forests mitigate this limitation by aggregating multiple trees and have become standard tools for identifying influential genes, epigenetic features, and expression patterns associated with DNA damage phenotypes (Aranguiz et al., 2025; Rowe et al., 2014). SVMs, particularly with radial-basis or polynomial kernels, are well suited for separating complex phenotypic classes in moderate-sized datasets, including those derived from mutational signatures (Patterson et al., 2023; Wagner et al., 2025). Recent advances in explainability methods, such as SHAP analysis, have further improved the transparency of these models (Coronnello and Francipane, 2022; Wagner et al., 2025). Collectively, these approaches remain indispensable for exploratory modeling and hypothesis generation in DNA damage research.

### 2.3 Applications to DNA damage and repair

Across transcriptomic, proteomic, and high-content imaging datasets, random forests, SVMs, and penalized logistic regression models have been applied to classify compound genotoxicity and elucidate modes of action (Acharjee et al., 2020; O’Brien et al., 2025; Thienpont et al., 2023). Feature-importance scores and BN edge strengths can reveal pathway-level perturbations, informing targeted follow-up experiments. Robust analytical pipelines typically incorporate batch correction, variance stabilization, nested cross-validation, and probability calibration to minimize false discoveries (Yamane et al., 2016; Zhang and Aires-de Sousa, 2007).

For variant classification in DNA repair genes (e.g., *BRCA1*, *ATM*, *XRCC1*), predictive performance improves when sequence-level features, evolutionary conservation, protein-domain annotations, and expression data are integrated. Logistic regression and SVMs remain competitive for binary pathogenicity prediction, while RFs and BNs are advantageous for capturing epistatic interactions and contextual modifiers. Importantly, calibrated probability outputs support downstream decision-making in experimental validation pipelines.

Despite substantial progress, several DNA repair mechanisms remain incompletely understood, particularly with respect to quantitative kinetics, cross-pathway crosstalk, and spatiotemporal regulation. Table 1 summarizes key outstanding gaps.

**TABLE 1:** This table critically examines recent computational approaches—especially AI and deep learning methods—used to investigate DNA damage and repair. We identified and summarized in this table existing limitations and highlighted areas where mathematical and computational insights are most urgently needed.

Repair pathway/Mechanism	Current knowledge	What’s Poorly understood/Needs computational work	Potential computational approaches
Double-strand break (DSB) Repair via homologous recombination (HR) and Non Homologous end joining (NHEJ)	Pathway choice depends on cell cycle, chromatin state, and DNA end structure	The decision-making process (how cells choose HR vs. NHEJ) remains only partially understood; stochastic influences and repair kinetics are complex	Probabilistic modelling, Bayesian inference, and agent-based simulations to quantify repair probabilities under different contexts
Base excision repair (BER)	Well-characterised enzymatically, but varies across tissues	The spatial organisation of BER enzymes in chromatin and the impact of oxidative stress dynamics are not fully mapped	Spatial-temporal stochastic simulations; ML-based 3D imaging analysis of enzyme recruitment
Nucleotide excision repair (NER)	Mechanistic steps known (damage recognition, excision, synthesis)	The global vs. transcription-coupled repair balance, and how chromatin compaction affects lesion recognition, are still unclear	Deep learning on chromatin accessibility datasets and molecular docking simulations
Mismatch repair (MMR)	Core proteins (e.g., MLH1, MSH2) are known	Error tolerance thresholds and mutation hotspots under different replication stress conditions lack quantitative models	Statistical and MLdriven mutation mapping, kinetic Monte Carlo models
Cross-talk between repair pathways	Multiple pathways can act on the same lesion type	Intergathway competition and signalling integration (e.g., PARP, ATM, ATR networks) are poorly modelled	Systems biology network models, dynamic Bayesian networks
Epigenetic and chromatin regulation	Epigenetic control over repair gene expression is known in part	The dynamic feedback loops between repair activity, chromatin remodelling, and transcription are not yet quantitatively understood	Integrative ML using ATAC-seg, ChIRseq, and transcriptomics data

#### 2.3.1 AI-driven discovery of DNA repair mechanisms (case study)

A central challenge in DNA damage research is understanding how cells integrate multiple contextual signals to determine repair pathway engagement (Her and Bunting, 2018; Sanchez et al., 2021; Kumari et al., 2025). Double-strand break repair, for example, involves competition between non-homologous end joining (NHEJ) and homologous recombination (HR), with pathway choice influenced by cell-cycle phase, chromatin state, damage complexity, and signaling dynamics. While individual pathway components are well characterized, their coordinated regulation remains difficult to resolve using reductionist approaches alone.

AI and machine-learning methods address this challenge by integrating heterogeneous datasets to infer regulatory dependencies and characterize repair kinetics at population scale (Pugliese et al., 2021; Zeitler et al., 2022). Bayesian network models have reconstructed probabilistic relationships between damage signaling markers, repair protein recruitment, and cellular context (Isci et al., 2014), identifying chromatin accessibility and replication-associated stress as key determinants of HR engagement—consistent with biological constraints on repair pathway choice (Chapman et al., 2012).

Mechanistic hypotheses generated by these approaches are supported by experimental validation. Perturbation of signaling kinases such as ATM, ATR, or DNA-PKcs leads to measurable shifts in repair pathway utilization, reflected in altered dynamics of canonical markers such as RAD51 and 53BP1 (Mladenov et al., 2019; Tomimatsu et al., 2009). Beyond pathway choice, machine-learning models have captured temporal patterns in γ H2AX foci resolution and repair protein turnover, revealing kinetic signatures associated with efficient versus stalled repair (Granzotto et al., 2024; Planck et al., 2024). Time-course irradiation experiments confirmed that these AI-derived profiles correspond to biologically distinct repair states (Zeitler et al., 2022).

Collectively, these studies demonstrate that AI-based approaches can contribute directly to mechanistic understanding of DNA damage and repair by integrating complex datasets, identifying regulatory dependencies, and guiding targeted experimental validation. Rather than replacing wet-lab experimentation, AI-driven models function as hypothesis-generation tools that augment traditional biological inquiry by revealing system-level properties that are otherwise difficult to discern.

## 3 Artificial intelligence modelling paradigms for DNA damage and repair

The application of AI to DNA damage and repair has given rise to a diverse set of modeling paradigms, each offering distinct advantages depending on the biological question under investigation. In this section, we focus on Bayesian networks (BNs), artificial neural networks (ANNs), and related hybrid approaches, not as competing methodologies but as complementary frameworks that address different aspects of DNA damage response (DDR) complexity.

Bayesian networks are particularly well suited to modeling DNA repair processes where causal structure, conditional dependencies, and interpretability are central, such as inferring pathway interactions or regulatory control points within the DDR (Needham et al., 2007). By contrast, artificial neural networks excel at capturing non-linear relationships in high-dimensional data, including imaging, transcriptomic, and multi-omics datasets, where predictive accuracy is prioritized over mechanistic transparency (Zhang et al., 2025). Recent developments have increasingly explored hybrid and probabilistic neural models, including Bayesian neural networks, which seek to combine the expressive power of deep learning with uncertainty quantification and biological interpretability. Such approaches are especially relevant in DNA repair research, where stochasticity, context dependence, and experimental uncertainty are inherent features of the system (Friedman et al., 2000; Isci et al., 2014; Dolan et al., 2015; Angelopoulos et al., 2022).

We begin by discussing applications of artificial neural networks, which have been widely adopted for predictive and simulation-based analyses of DNA damage and repair, before considering probabilistic and causal modeling approaches.

### 3.1 Artificial neural networks to analyse DNA damage and repair

Artificial neural networks (ANNs) are capable of examining DNA repair processes by assimilating complex biological information to forecast outcomes, assess genetic variation, and identify previously unrecognised repair components or pathways. ANNs have been widely applied to the analysis of high-throughput microscopy images (Tandon et al., 2022), enabling real-time observation of DNA repair dynamics and automated identification of proteins involved in the DNA damage response (You et al., 2022; Qiu et al., 2024). Additional applications include predicting the functional impact of genetic variants on DNA repair proteins, identifying candidate therapeutic compounds, and modeling interactions between DNA repair and other cellular processes, such as autophagy. A recent example is the comprehensive simulation framework GANDALF (Generative ANsatz for DNA damage evaluation and Forecast), developed by Sciuto et al. (2025) which integrates neural network–based regression with multiscale radiation damage simulations. GANDALF links micro-scale radiation parameters, such as linear energy transfer (LET) tracks, to nano-scale DNA damage outcomes, including double-strand break formation. By learning this mapping, the framework substantially reduces the computational cost of nano-scale simulations, eliminating the need to explicitly simulate damage at every spatial scale.

### 3.2 Bayesian networks and deterministic neural networks

Bayesian networks (BNs) are explainable AI models capable of representing causal relationships between nodes. In understanding the intricate relationships among various variables in biological contexts, they can be developed from observed data and serve as a helpful visual and computational tool in investigating these relations.

**FIGURE 3:**
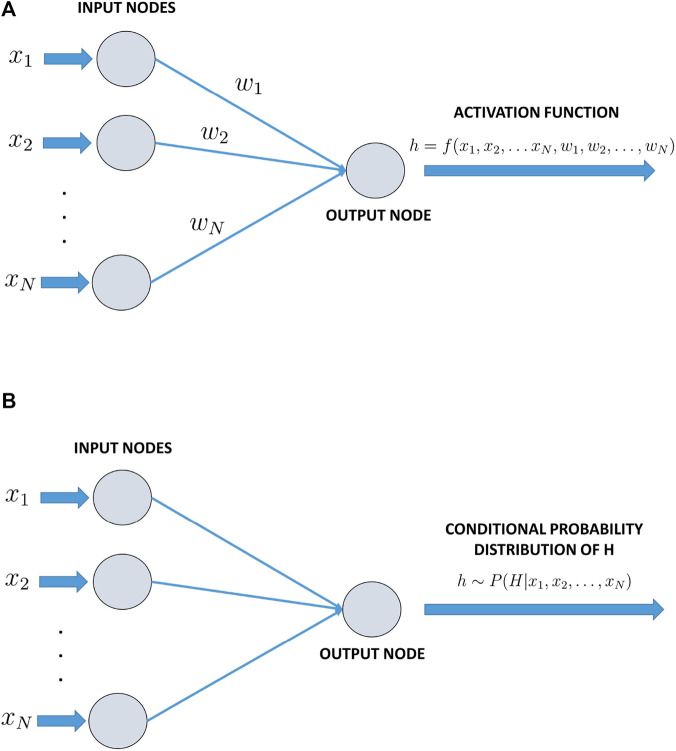
**(a)** A deterministic neural network, where f in Equation 1 is a deterministic function. **(b)** A Bayesian neural network, where f of Equation 1 is a sampling operator (see Equation 2).

BNs are probabilistic graphical models in which edges represent causal relationships between nodes. Specifically, BNs are directed acyclic graphs (DAGs), similar in structure to neural networks, that encode dependencies among variables and enable the updating of conditional probabilities based on *prior* knowledge and newly observed data (ElKalaawy and Wassal, 2015). The structure of a BN is defined by the quantitative variables associated with each node, their marginal probabilities, and the conditional probability distributions of nodes with incoming edges (i.e., nodes with parent nodes). For instance, in the context of biochemical or protein–protein interaction networks, the variables associated with nodes may represent the concentrations of molecular species. Similarly, in gene regulatory networks, nodes may correspond to gene expression levels. When the interaction network being modeled is of manageable size, it is often feasible to specify both the marginal and conditional probability distributions explicitly. These conditional probabilities link the values of parent nodes to those of their child nodes and capture the probabilistic relationships governing the system (Aggarwal, 2021). Of particular interest in the context of this article is the relationship between BNs and neural networks. While neural networks employ deterministic functions in directed acyclic graph settings, BNs utilise functions that efficiently draw values from probability distributions. Let us consider a neural network like in Figure 3a, where a node with a variable h receives the incoming values $x_{1},x_{2},\ldots ,x_{N}$ . In that scenario, the neural network calculates a deterministic function f(⋅) such that

$$h=f\left(x_{1},\ldots ,x_{d}\right).$$ (1)

Examples of such functions, known as *activation functions*, may include the linear operator, the sigmoid operator, or a composition of the two operators. In the case of a BN as in Figure 3b, the function f is defined as a sampling operator, wherein the value h is sampled from a conditional probability distribution $P\left(H|x_{1},\ldots ,x_{N}\right)$ of the random variable H , given its inputs Aggarwal (2021), i.e.,

$$h∼P\left(H|x_{1},\ldots ,x_{N}\right).$$ (2)

A Bayesian network can therefore be regarded as a stochastic (or randomized) network, since it may generate different outputs for the same input when the inference or sampling process is repeated. This variability arises because each output corresponds to a realization drawn from a probability distribution, rather than the evaluation of a deterministic function such as $f\left(x_{1},\ldots ,x_{d}\right)$ .

Traditional Bayesian networks assume that probability distributions are fully specified *a priori* in a manner tailored to the application domain. This assumption becomes increasingly impractical as the scale and complexity of the network grow. In particular, joint and conditional probability distributions become progressively more difficult to define as the number of parent nodes associated with a given node increases. Contemporary machine learning approaches extend beyond this classical formulation by enabling probability distributions to be learned directly from data. When learning mechanisms are incorporated, Bayesian networks evolve into a broad class of probabilistic graphical models grounded in machine learning principles (Sammut and Webb, 2010; Aggarwal, 2021; Lapenna and De Bacco, 2025; Hua et al., 2025).

BNs also satisfy the local Markov property, commonly referred to as the *Markov property*. According to this property, the value of a variable at a given node—denoted here by h —depends only on its direct parent nodes and not on more distant ancestors that are not directly connected to it. Consequently, a node is conditionally independent of its non-descendant ancestors given the values of its immediate parents. The Markov property is widely used in sequence-based models in machine learning, such as hidden Markov models (HMMs) (Yoon, 2009; Von Bülow et al., 2025; Xu, 2025).Bayesian networks provide a natural generalization of these ideas, allowing dependencies that are not restricted to linear or sequential structures but instead form arbitrary directed acyclic graphs. To illustrate this idea, consider a Bayesian network in which there is a directed edge from variable $h_{1}$ to $h_{2}$ , and from $h_{2}$ to $h_{3}$ , but no direct edge from $h_{1}$ to $g_{3}$ . In this case, the local Markov property implies that:

$$P\left(h_{3}|h_{2},h_{1}\right)=P\left(h_{3}|h_{2}\right)$$ (3)

**FIGURE 4:**
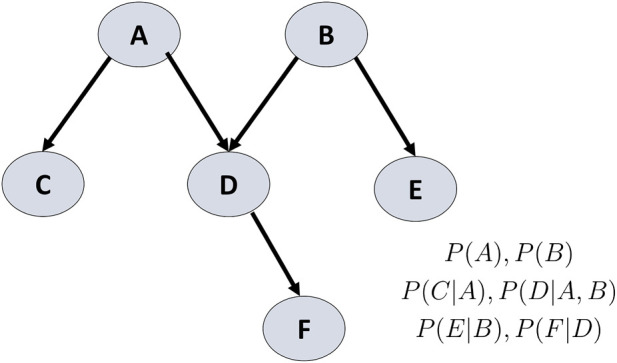
An example of a Bayesian network. A directed edge from node X to node Y indicates that X has a direct influence on Y, quantified by the conditional probability of Y given X. That is, it represents the probability that the quantitative variables associated with Y take specific values (e.g., Y=y ), when the variables associated with X take specific values (e.g., X=x ). In mathematical terms, a Bayesian network represents a factorization of a joint probability distribution into an acyclic set of conditional dependencies, which are visually depicted as a directed acyclic graph (Hammond and Smith, 2025). This graphical structure directly corresponds to the mathematical expression of the joint probability distribution. Specifically, the joint probability distribution $P\left(A,B,C,D,E,F\right)$ , which represents the probability of all variables simultaneously occupying particular states, factorizes as the product of the conditional probabilities of each node given its parent nodes:. $P\left(A,B,C,D,E,|F\right)=P\left(A\right)\cdot P\left(B\right)\cdot P\left(C|A\right)\cdot P\left(D,A,B\right)\cdot P\left(E|B\right)\cdot P\left(F|D\right)$ .

However, if a direct edge were present from $h_{1}$ to $h_{3}$ , the requirement in Equation 3 would no longer hold. The local Markov property holds in Bayesian networks because the value of each variable is generated as a sampling function that depends exclusively on the values of its directly connected parent nodes. While indirect ancestors influence the system indirectly by affecting the values of parent nodes, a given node requires only its immediate parents as inputs. Each node in a Bayesian network is associated with a conditional probability table (CPT) containing $2^{k}$ probability values, where k denotes the in-degree of the node—that is, the number of its parent nodes. Thus, each node is associated with a table of probability values that represents a joint probability distribution over the node and its parent variables, rather than a single conditional probability assigned to each individual edge (see Figure 4).

### 3.3 Applications and perspective on Bayesian neural networks

The usefulness of BNs in systems biology—and particularly in DNA and gene biology—has been recognized for more than 15 years, as evidenced by the extensive scientific literature from that period, which continues to influence current research [see, for example, (Hammond and Smith, 2025; Saint-Antoine and Singh, 2020; Agrahari et al., 2018; Su et al., 2013; Larjo et al., 2013; Needham et al., 2007; Wilkinson, 2006; Husmeier, 2005)]. It is now widely acknowledged that many diseases have a genetic component but do not follow simple Mendelian patterns of dominant or recessive inheritance. Instead, these conditions are likely to arise from the combined effects of multiple genes together with one or more environmental risk factors. Such complex phenotypic traits are characterized by substantial variability and uncertainty, as both the number and nature of the underlying interactions are difficult to identify using traditional analytical approaches. Nevertheless, uncovering the genetic basis of disease and quantifying the relative contribution of environmental influences are essential steps toward the development of effective personalized medicine frameworks.

A comprehensive review by Su et al. (2013) highlights how machine learning methods—particularly BNs—can help disentangle the intricate relationships among genes, environmental factors, and disease phenotypes. BNs provide a multivariate modeling framework capable of simultaneously capturing gene–gene interactions (epistasis) and gene–environment interactions, while also incorporating diagnostic information derived from clinical or physiological variables. In addition, BNs naturally support prognostic modeling: once constructed, a network can compute the probability that an individual with a specific genotype and environmental exposure will exhibit a given phenotype. Beyond genetic epidemiology, BNs have been applied extensively in causal inference and probabilistic prediction across diverse domains, including medical diagnosis, forensic science, crime and terrorism assessment, and environmental conservation. In bioinformatics, they have been used to analyze gene expression data, reconstruct protein signaling networks, predict protein–protein interactions, perform pedigree analysis, conduct genetic epidemiological studies, and evaluate the prognostic value of microsatellite markers in cancer recurrence. Overall, BNs offer significant potential for elucidating the genetic and environmental determinants of disease. However, as emphasized by Su et al. (2013), despite their effectiveness in genetic disease analysis and prognostic modeling, BNs face several conceptual and computational challenges. These include the proper handling of continuous variables and latent (unmeasured) factors, the explicit incorporation of *prior* biological knowledge, and the rigorous evaluation and communication of inference robustness under varying assumptions and data representations. Notably, many of these theoretical and computational issues remain only partially resolved. The increasing integration of AI into data modeling and simulation has therefore raised a natural question: can artificial neural networks (ANNs) help overcome some of the intrinsic limitations of classical Bayesian networks? BNs and ANNs represent two distinct classes of graphical models widely used in machine learning and AI. Although both are designed to model complex systems and generate predictions, they differ substantially in their theoretical foundations, computational mechanisms, and areas of application. The principal differences between these approaches are summarized in Table 2.

**TABLE 2:** Main differences between Bayesian Networks and Artificial Neural Networks. Several properties highlighted in this comparison are particularly relevant for the study of DNA damage and repair mechanisms. For example, the interpretability of Bayesian networks facilitates the analysis of pathway crosstalk and regulatory dependencies within DNA damage response (DDR) networks. Conversely, Bayesian neural networks provide explicit uncertainty quantification, which is valuable for assessing confidence in predictions related to DNA damage severity, repair efficiency, or clinical risk. Deterministic neural networks remain highly effective for large-scale data-driven tasks such as image-based detection of DNA damage from comet assay or fluorescence microscopy data.

Feature	Bayesian networks	Artificial neural networks
Interpretability	Bayesian networks illustrate and analyse clear probabilistic connections and dependencies among variables via a directed acyclic graph, offering interpretable models and estimates of uncertainty	Artificial Neural Networks employ a non-transparent network of linked neurons to grasp intricate patterns straight from data for purposes such as classification and prediction, but their internal architecture does not naturally depict statistical connection
Data requirements	Can integrate specialized expertise, ideal for limited datasets	Generally need substantial quantities of annotated data
Adaptability	Adaptable and modular, can update parts independently	Less adaptable, frequently need retraining on updated data to adjust to modifications
Performance and scalability	More computational resource-efficient, simpler to scale	Typically outperform Bayesian Networks in activities demanding high precision and in case of complex, non-linear relationships
Uncertainty	Bayesian networks naturally embody uncertainty via probability distributions	Typically provide point estimates

ANNs can be used to overcome several limitations of BNs, while Bayesian networks themselves can benefit from Bayesian inference when incorporated into so-called Bayesian artificial neural networks (BNNs). From our perspective, BNNs are particularly well suited to the study of DNA repair mechanisms and DNA damage assessment. This suitability arises from their ability to explicitly represent uncertainty through probability distributions while maintaining the scalability and expressive power of deep learning models. In many contexts, BNNs may outperform classical Bayesian networks while retaining probabilistic interpretability. To clarify the rationale behind our perspective and for the reader’s convenience, we provide a brief overview of BNNs below.

ANNs are widely regarded as powerful function approximators. Their effectiveness stems from the high adaptability of a large number of model parameters—weights and biases—that are learned from data using gradient-based optimization. When sufficient data are available, neural networks excel at approximating complex input–output relationships, making them highly effective in tasks such as speech recognition, image classification, and other data-intensive AI applications. However, this flexibility also introduces a major limitation: neural networks are particularly prone to overfitting. Overfitting occurs when the learning algorithm optimizes model parameters too closely to the training data, resulting in degraded performance on unseen data. Modern deep neural networks often contain millions of parameters, which substantially increases the risk of overfitting, as the model can effectively memorize training data, including noise and spurious correlations. This issue is exacerbated when training datasets are limited, imbalanced, or unrepresentative. As a result, deep neural networks may achieve low training error while exhibiting high generalization error. A critical consequence of this behavior is that standard deep neural networks tend to produce confident predictions even when presented with data outside their training distribution—situations in which they should instead express uncertainty or acknowledge insufficient knowledge. This limitation is particularly problematic in high-stakes domains such as medicine and toxicology, including DNA damage assessment, where incorrect but confident predictions can have serious consequences. To address this limitation, uncertainty can be incorporated directly into the learning process through stochastic neural networks. These models introduce stochasticity either in the network weights or in the activation functions, enabling the representation of multiple plausible models θ , each associated with a probability distribution P(θ) (Belli et al., 1999; Geretti and Abramo, 2011; Yu et al., 2021). Uncertainty is quantified by comparing predictions or forecasts obtained from different sampled parameterizations θ (Jospin et al., 2022)—where agreement among models indicates low uncertainty, while divergence signals high uncertainty. As summarized by Jospin et al. (2022) this process can be expressed as:

$$\begin{array}{cc} & \theta ∼P\left(\theta \right)\\ & y=\phi_{\theta }\left(x\right)+\epsilon \end{array}$$ (4)

where ϵ represents random noise, reflecting the fact that the function ϕ provides only an approximation of the true data-generating process. A BNN is therefore defined as a probabilistic neural network trained using Bayesian inference. The construction of a BNN begins with the selection of a neural network architecture, which serves as a functional model. A stochastic model is then specified by choosing a prior distribution over the model parameters P(θ) and a likelihood function P(y∣x,θ) , representing beliefs about the model’s forecasting capacity. According to Jospin et al. (2022), the parametrization of the model in Equation 4 may be viewed as a hypothesis H , and the training set, data D . The choice of a BNN’s stochastic model is somehow equivalent to the choice of a loss function when training a point estimate neural network. Using the notation of Jospin et al. (2022), let the model parameters be denoted by θ , the training dataset by D , the inputs by $D_{x}$ , and the outputs by $D_{y}$ . Under the assumption of independence between parameters and inputs, Bayes’ theorem yields the posterior:

$$P\left(\theta |D\right)=\frac{P\left(D_{y}|D_{x},\theta \right)P\left(\theta \right)}{\int_{\theta }P\left(D_{y}|D_{x},\theta^{\prime }\right)P\left(\theta^{\prime }\right)d\theta^{\prime }}\propto P\left(D_{y}|D_{x},\theta \right)P\left(\theta \right).$$ (5)

The Bayesian posterior for intricate models like ANNs represents a high-dimensional and significantly non-convex probability distribution. This complexity renders it a challenging issue to compute and sample it with traditional techniques, particularly since obtaining the evidence $\int_{\theta }P\left(D_{y}|D_{x},\theta^{\prime }\right)P\left(\theta^{\prime }\right)d\theta^{\prime }$ is arduous. To tackle this issue, Jospin et al. (2022) proposed two main strategies: (i) Markov chain Monte Carlo and (ii) variational inference (Blei et al., 2017). When employing a BNN for forecasting, the probability distribution P(y∣x,D) , referred to as the *marginal* and which measures the model’s uncertainty regarding its prediction, is especially significant. Given P(θ∣D) , it is possible to calculate P(y∣x,D) as:

$$P\left(y|x,D\right)=\int_{\theta }P\left(y|x,\theta^{\prime }\right)P\left(\theta^{\prime }|D\right)d\theta^{\prime }$$ (6)

where in practice P(y∣x,D) is sampled indirectly from Equation 4. Indeed, a set of weights $\theta_{i}$ is sampled from the posterior and used to compute a series of possible outputs $y_{i}$ , which corresponds to samples from the marginal (Jospin et al., 2022).

When performing regression, the procedure commonly adopted to summarize the predictions of a BNN is the model average (Gal and Ghahramani, 2015):

$$\hat{y}=\frac{1}{\left|\Theta \right|}\underset{\theta_{i}\in \Theta }{\sum }\phi_{\theta_{i}}\left(x\right).$$ (7)

To measure uncertainty, the covariance matrix can be calculated as shown below:

$$\underset{\gamma |x,D}{\sum }=\frac{1}{|\Theta |-1}\underset{\theta_{0}\in \Theta }{\sum }\left(\phi_{\theta_{0}}\left(x\right)-\hat{y}\right){\left(\phi_{\theta_{0}}\left(x\right)-\hat{y}\right)}^{⊤}.$$ (8)

In classification tasks, the average prediction from the model provides the relative likelihood of each class, serving as an indicator of uncertainty:

$$\hat{p}=\frac{1}{\left|\Theta \right|}\underset{0,\in \Theta }{\sum }\phi_{0}\left(x\right).$$ (9)

The final prediction is taken as the most likely class:

$$\hat{y}=\underset{i}{\text{argmax }}p_{i}\in \hat{p}.$$ (10)

Within the framework defined by the Equations 5–10, BNNs function as discriminative models, meaning models that seek to reconstruct a target variable y from observed data x while simultaneously quantifying uncertainty.

The relevance of BNNs to DNA damage assessment is substantial. By modeling uncertainty in network parameters, BNNs provide both predictions and confidence estimates, which are critical in genomics and toxicology. BNNs have been shown to classify compounds according to toxic effects, analyze complex toxicological datasets, and quantify DNA damage in imaging data from techniques such as *comet assays*, often with improved robustness compared to conventional neural networks (Pantic et al., 2023). Early work by Sharma et al. (Sharma et al., 2011) demonstrated the utility of Bayesian classifier networks for predicting mutagenicity, outperforming traditional feedforward neural networks on the Bursi mutagenicity dataset (Kazius et al., 2004). The findings indicated that the Bayesian classifier exhibited superior overall prediction accuracy (66.61%) compared to the traditional neural network (59.72%), yet it performed less accurately than several other machine learning models, including those utilizing support vector machines. More recent studies, such as that of Semenova et al. (2020) have shown that BNNs can outperform classical statistical models in predicting drug-induced liver injury, even when trained on relatively small datasets. These findings highlight the potential of BNNs as efficient classifiers of mutagenicity and molecular lesions, suggesting their broader applicability to DNA damage classification using omics data.

A notable advancement in this direction is the work of Joshi and Dhar (2022) who applied BNNs to the classification of cancer types and subtypes using transcriptomic data. Transcriptomics is particularly well suited for this task, as diverse genomic and epigenomic alterations often converge on shared gene expression programs. However, such data are high dimensional, noisy, and characterized by complex dependencies, posing significant challenges for traditional modeling approaches. While deep learning methods are well equipped to capture nonlinear patterns in transcriptomic data, point-estimate neural networks can produce overconfident predictions, especially in settings with limited data or class imbalance. In contrast, BNNs provide uncertainty estimates for individual predictions, which is essential for clinical decision-making, particularly when classifying individual patient samples. As molecular subtyping increasingly guides therapeutic strategies, especially in cancers such as breast cancer (Weigelt et al., 2008), the need for reliable and interpretable classification tools continues to grow. Machine learning approaches—including BNNs—offer significant advantages over conventional clinical and histological classification methods, which often suffer from limited prognostic accuracy (Ben-Ishay, 2013). A broad range of computational methods has already demonstrated value in cancer classification, biomarker discovery, and therapy selection (Sinkala et al., 2020; Sanders et al., 2022; Yan et al., 2025). Within this landscape, BNNs represent a particularly promising direction due to their ability to integrate predictive performance with principled uncertainty quantification.

#### 3.3.1 BNNs in DNA repair modeling and damage assessment

The growing complexity of DNA damage and repair research—driven by high-throughput omics technologies, advanced imaging platforms, and increasingly heterogeneous clinical and experimental datasets—demands computational frameworks that are both expressive and reliable, capable of integrating diverse sources of biological information and capturing complex regulatory dependencies (Mohammad et al., 2026).

In this context, BNNs offer a compelling synthesis of scalability, predictive power, and principled uncertainty quantification. Unlike classical Bayesian networks, which can struggle with dimensionality and rigid structural assumptions, and unlike standard deep neural networks, which often produce overconfident predictions, BNNs provide a balanced framework particularly well suited to the biological and clinical challenges inherent to DNA repair research.

DNA repair pathways, including BER, NER, HR, and NHEJ, are inherently multistep, stochastic, and context dependent. Their regulation involves complex, nonlinear interactions among genes, proteins, epigenetic states, and environmental stressors (Bryant et al., 2005; Ciccia and Elledge, 2010; Farmer et al., 2005; Groelly et al., 2022; Jackson and Bartek, 2009; Lam, 2022; Lord and Ashworth, 2012; Tong et al., 2024). Modeling such systems requires computational approaches that can integrate diverse data modalities while explicitly accounting for uncertainty and partial observability.

BNNs are well positioned to address these requirements. By placing probability distributions over network parameters, BNNs can capture uncertainty arising from limited data, experimental noise, and biological variability—features that are intrinsic to pathway-level modeling. When applied to omics datasets, BNNs can learn latent representations of pathway activity while simultaneously providing confidence estimates for inferred relationships or predicted repair outcomes. This is particularly important when attempting to infer pathway dysregulation in disease states, where data are often sparse or biased toward specific experimental conditions. Moreover, BNNs can be integrated with mechanistic knowledge derived from established DNA repair models. Priors over network parameters can encode known pathway constraints, such as the involvement of specific proteins in lesion recognition or repair complex assembly, thereby guiding learning toward biologically plausible solutions. In this sense, BNNs act as a bridge between purely data-driven deep learning approaches and mechanistic, hypothesis-driven modeling frameworks.

#### 3.3.2 Integration with imaging-based DNA damage assessment

One of the most immediate and impactful applications of BNNs lies in the analysis of imaging data from DNA damage assays, such as *comet assays* (Cordelli et al., 2021), γ H2AX immunofluorescence (Reddig et al., 2018), and live-cell microscopy (Heemskerk et al., 2023). These techniques generate large volumes of high-dimensional image data, whose interpretation is often time-consuming and subject to operator variability. Deep learning has already demonstrated substantial gains in automating these analyses, but standard neural networks provide limited insight into the reliability of individual predictions.

BNNs can directly address this limitation by associating each prediction with a measure of uncertainty. In imaging workflows, this capability is crucial. For example, ambiguous or low-quality images, rare damage phenotypes, or experimental conditions underrepresented in training data can be flagged automatically through elevated predictive uncertainty. This enables more robust downstream analysis and supports human-in-the-loop decision-making, where expert review is focused on uncertain or high-risk cases. Furthermore, uncertainty-aware image analysis is particularly valuable in genotoxicity testing and regulatory toxicology. When evaluating the DNA-damaging potential of novel compounds, especially under conditions where experimental replication is limited, BNN-based image classifiers can provide not only predictions but also confidence intervals, facilitating more transparent risk assessment and potentially reducing reliance on animal testing.

#### 3.3.3 Toward multiscale and translational applications

A key advantage of BNNs is their compatibility with multiscale modeling approaches. Imaging-derived features, molecular readouts, and clinical metadata can be jointly incorporated within a unified probabilistic framework, allowing damage phenotypes observed at the cellular level to be linked to pathway-level dysfunctions and, ultimately, to organism-level outcomes Choudhary et al. (2025); Phan et al. (2012); Al-Zoghby et al. (2025). This integrative capacity is essential for translational applications, such as predicting patient-specific repair deficiencies or stratifying responses to DNA-damaging therapies. From a clinical perspective, the ability of BNNs to quantify predictive uncertainty is particularly important. In precision medicine settings, where treatment decisions may depend on inferred repair capacity or predicted sensitivity to genotoxic agents, overconfident but incorrect predictions can have serious consequences. The capacity of BNNs to quantify predictive uncertainty is especially valuable for addressing several unresolved challenges in DNA damage and repair research. For example, when predicting the pathogenicity of rare variants in DNA repair genes such as *BRCA1*, available datasets are often limited and highly imbalanced. That is to say, in such contexts, a deterministic neural network typically produces a single classification outcome (e.g., pathogenic vs. benign), whereas a BNN produces a predictive probability distribution reflecting the model’s confidence in the classification. This probabilistic output can assist clinicians and researchers by indicating when predictions are reliable and when additional experimental validation or clinical interpretation may be required. A similar advantage arises in the analysis of microscopy-based assays used to quantify DNA damage, such as comet assays or fluorescence imaging of DNA repair foci. In these workflows, image classification models are typically trained to assign damage levels based on morphological features extracted from microscopy images (see Section 5 for more information on DNA damage detection from images). A BNN-based classifier can provide not only the predicted damage category but also an associated uncertainty estimate. For instance, when analysing a comet image the model may assign probabilities such as 40% to damage class 3, 30% to class 4, and 30% to class 2. Such low-confidence predictions can be automatically flagged within a human-in-the-loop analysis pipeline, prompting expert review of ambiguous images. This strategy improves both the reliability and transparency of automated DNA damage assessment, particularly in high-throughput screening or clinical contexts where decision-making requires quantifiable confidence levels. BNNs therefore offer a mathematically grounded framework for expressing predictive uncertainty, supporting safer and more interpretable AI-assisted decision-making in the analysis of DNA damage and repair. However, despite their promise, several challenges remain before BNNs can be routinely deployed in DNA repair research and clinical workflows. These include the computational cost of Bayesian inference in large-scale networks, the need for standardized benchmarks in DNA damage imaging and pathway prediction, and the development of user-friendly tools that integrate BNN outputs into existing bioinformatics pipelines. Advances in approximate inference methods, scalable variational techniques, and hybrid models that combine mechanistic constraints with probabilistic deep learning are likely to play a key role in addressing these challenges. BNNs represent a powerful and versatile framework for advancing the computational study of DNA damage and repair. By unifying deep learning with probabilistic reasoning, BNNs enable robust modeling of complex biological systems while explicitly accounting for uncertainty—an essential requirement for reliable inference in both research and clinical domains.

## 4 Recurrent neural networks

**FIGURE 5:**
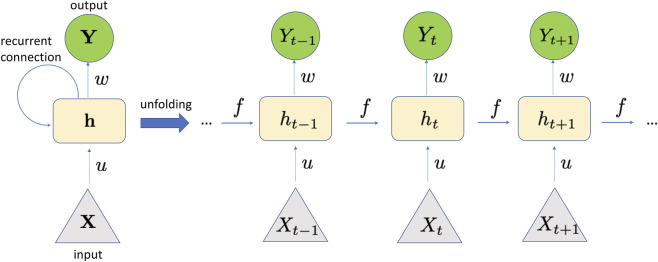
The structure of an RNN can be viewed as an expanded form of a standard feed-forward neural network. An RNN can be seen as a sequence of feed-forward networks, where each layer is linked to both the prior and subsequent layer via shared weights and biases. The ability of RNNs to use information from prior inputs to influence the current input and output distinguishes them from standard deep neural networks, which treat inputs and outputs as independent. The network contains two groups of weights: one for the inputs and one for the hidden state vector. During execution, the hidden state—shaped by previous inputs—together with the current input, determines the output. The output is calculated as in Equation 13.

Classification tasks can be effectively performed by recurrent neural networks (RNNs) when working with sequence and time-series data (Ergen and Ceyani, 2018; Lecca, 2024). The RNN is a type of neural network where the output from the previous step serves as input for the current step (Figure 5). Conventional neural networks possess separate inputs and outputs. Nonetheless, for example, when anticipating the next word in a sentence, it is necessary to remember the earlier words. Consequently, RNN was developed to address this issue by utilizing a hidden layer. The primary and most crucial feature of an RNN is its *hidden state*, which holds certain information regarding a sequence. The state is referred to as *memory state* because it holds the previous input to the network. The hidden state utilizes identical parameters for every input and executes the same operation on all hidden inputs or layers to produce the output.

The RNN consists of several fixed activation function units, one designated for each time step. Every unit contains a concealed state. This concealed state indicates the previously acquired knowledge that the network holds at a specific time step. This concealed state is modified at every time step to represent any alterations in the network’s understanding of the past. The hidden state is modified using the subsequent recurrence relation:

$$h_{t}=f\left(h_{t-1},X_{t}\right)$$ (11)

where, $h_{t}$ is the current state, $h_{t-1}$ is the previous state, and $X_{t}$ is the input state. The function f in Equation 11 is applied as follows:

$$h_{t}=f\left(w_{hh}h_{t-1}+w_{xh}X_{t}\right)$$ (12)

where, $w_{hh}$ is the weight at recurrent neuron, and $w_{xh}$ weight at input neuron.

Finally, the output $Y_{t}$ is

$$Y_{t}=w_{hy}h_{t}$$ (13)

where $w_{hy}$ is the weight at output layer, and *h_t_* is given by Equation 12.

### 4.1 Applications and perspective on recurrent neural networks

RNNs are well suited for modeling DNA repair processes, as DNA sequences constitute sequential data and RNNs possess internal memory mechanisms (feedback loops) that allow them to handle sequences, retain contextual information, and generate predictions. RNN architectures such as long short-term memory (LSTM) networks and gated recurrent units (GRUs) have been applied to tasks including the classification of DNA variants, prediction of the behavior of repair-associated proteins, and simulation of complex molecular dynamics in DNA repair pathways.

**FIGURE 6:**
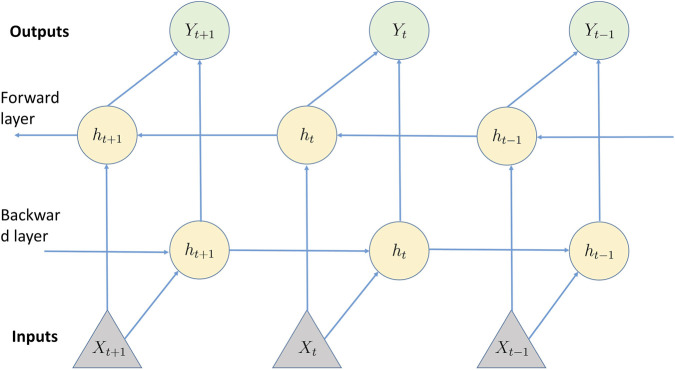
A Bidirectional Recurrent Neural Network (BRNN) extends the conventional RNN by processing sequential data in both forward and reverse directions. This allows the network to leverage both historical and future context when generating predictions. As in a conventional RNN, a BRNN advances through the sequence, updating the hidden state based on the current input and the previous hidden state at each time step. The key distinction is the presence of a backward hidden layer that analyzes the sequence in reverse, updating the hidden state using information from the next time step. By incorporating both past and future context, BRNNs improve accuracy over unidirectional RNNs, with predictions generated from the combined outputs of the forward and backward layers.

A bidirectional recurrent neural network (BRNN) is a variant of RNNs designed to enhance the capabilities of standard RNN architectures by analyzing sequential data in both forward and backward directions (Figure 6). This design enables the network to exploit information from both preceding and subsequent contexts, which can be particularly advantageous for tasks in which bidirectional context is essential, such as time-series analysis.

A seminal article published in 2019 by Liu et al. (2019), proposing the use of RNNs—and in particular BRNNs—for the detection of modified DNA bases, helped pave the way for the application of artificial neural networks in the detection and evaluation of DNA damage. The authors focused on DNA base modifications, including C5-methylcytosine (5 mC) and N6-methyldeoxyadenosine (6 mA), which are key epigenetic marks. Traditional methods such as short-read bisulfite sequencing and long-read PacBio sequencing exhibit fundamental limitations in detecting DNA modifications. To address this, Liu et al. (2019) leveraged raw electrical signal data generated by Oxford Nanopore Technologies (2025) long-read sequencing to develop DeepMod, a BRNN architecture incorporating LSTM units for the identification of DNA modifications. Following the work of Liu et al. (2019), several studies have proposed RNN- and BRNN-based approaches for investigating DNA damage and modification status (Ni et al., 2019; Namuduri et al., 2019; Sun et al., 2023; Wan et al., 2022).

An additional notable study employing a bidirectional neural architecture was introduced by Yu et al. (2022). In this work, the authors developed a method termed 4mCi6mA-BGC for predicting DNA modification sites corresponding to 4mC and 6 mA. Their approach integrated multiple machine learning techniques, including elastic net feature selection (Chamlal et al., 2024) and a hybrid deep learning architecture composed of a bidirectional GRU (BiGRU) and a convolutional neural network (CNN). Several feature extraction methods—binary encoding, k-mer nucleotide frequencies, pseudo k-tuple nucleotide composition (PseKNC), dinucleotide-based autocovariance (DAC), and MonoDiKGap—were combined to convert DNA sequences into numerical representations suitable for machine learning. The elastic net method was then applied to eliminate redundant or non-informative features, yielding an optimal feature subset that was subsequently used as input to the BiGRU–CNN model. The results reported by Yu et al. (2022) demonstrated that the 4mCi6mA-BGC framework significantly improved prediction accuracy for 4mC and 6 mA modification sites.

As illustrated by the study of Yu et al. (2022), combining BRNNs with CNNs opens new perspectives for computational approaches in DNA damage analysis. The BiGRU–CNN architecture leverages CNNs for spatial feature extraction and BiGRUs for modeling temporal dependencies, enabling simultaneous analysis of both the “where” (spatial) and “when” (temporal) aspects of sequential biological data. This dual capability makes such architectures particularly effective and accurate for forecasting and classification tasks involving complex biological sequences.

Beyond epigenetic applications, RNN-based models can also be employed to address questions related to the temporal progression of DNA repair signaling and pathway activation. One biologically relevant problem, for instance, is predicting whether an acute DNA double-strand break will be efficiently resolved or whether damage signaling will persist, potentially leading to cell-cycle arrest or apoptosis. To tackle this problem, RNN or LSTM models can be trained on longitudinal datasets comprising time-series measurements such as γ H2AX foci intensity, recruitment kinetics of repair factors (e.g., p53 and Mdm2), and cell-cycle status following genotoxic stress. By learning temporal dependencies across these sequences, such models can infer how early signaling dynamics influence downstream repair outcomes and cell fate decisions.

RNNs have previously been applied to model oscillatory dynamics in DNA damage response (DDR) systems. A notable example is the modeling of the p53–Mdm2 feedback loop, a key regulatory motif activated following DNA damage (Ling et al., 2013). In this study, an RNN was used to represent the temporal dynamics of p53 and Mdm2 concentrations, capturing their oscillatory behavior while also enabling parameter estimation from sparse experimental data.

Beyond recurrent architectures, recent advances in sequence modeling suggest that transformer-based neural networks may offer additional advantages for studying DNA damage and repair mechanisms. Transformers employ attention mechanisms that enable models to capture long-range dependencies within genomic sequences, allowing the network to identify regulatory interactions between distant genomic regions, as demonstrated by recent deep-learning models of gene regulation and genome function (Avsec et al., 2021; Ji et al., 2021). This capability may be particularly relevant for modeling chromatin organization and regulatory interactions that influence DNA repair pathway choice and efficiency. Indeed, long-range chromatin contacts, epigenetic modifications, and transcriptional activity are known to modulate the accessibility of damaged DNA sites and the recruitment of repair factors (Aymard et al., 2014; Clouaire and Legube, 2019). Attention-based models could therefore complement recurrent neural networks by capturing regulatory dependencies that extend beyond the immediate sequence context. More recently, deep learning approaches incorporating recurrent architectures, attention mechanisms, and other sequence-aware models have been proposed to predict aspects of DNA damage induction and repair from sequential molecular features, further demonstrating the feasibility and promise of such methods in this domain (Alsharaiah et al., 2022; Liu et al., 2022). Importantly, predictions generated by these models can be experimentally evaluated using time-course irradiation assays, live-cell imaging of repair foci formation and resolution, or perturbations of key DDR regulators. These experimental validations help determine whether temporally learned patterns reflect meaningful biological states rather than modeling artefacts.

## 5 DNA damage detection from images

Research on DNA damage has been greatly supported by advanced DNA imaging systems, that is, devices capable of visualizing the delicate DNA structure, combined with biological preparation procedures and computer vision algorithms enabling the analysis of DNA morphology. DNA observation requires specialized instruments, such as fluorescence microscopes (Goodhew and Humphreys, 2000), electron microscopes (Breese et al., 2007), atomic force microscopes (Main et al., 2021), focused-ion beam scanning systems (Goldstein, 2012), and nanofluidic devices (Müller and Westerlund, 2017). Unlike conventional light microscopes, which cannot resolve structures as small as the DNA helix (approximately 0.01 μ m in width), these advanced systems can achieve resolutions on the order of 100 p.m., allowing visualization of fine DNA structural components. Their high magnification capability is achieved by probing the sample with beams of subatomic particles or charged atoms, which interact with the sample and produce an image based on the resulting signals. Fluorescence microscopy visualizes DNA within cells by detecting light emitted from fluorescent dyes introduced into cell-containing solutions. Electron microscopy techniques—such as transmission electron microscopy (TEM), scanning electron microscopy (SEM), and atomic force microscopy (AFM)—utilize beams of electrons or atoms, focused through magnetic lenses, to detect fine structural details of DNA, including molecules and chromosomes. Ion-beam scanning systems operate similarly to electron microscopes but use ions instead of electrons, or in some cases both. Atomic force microscopes provide three-dimensional topographic information: a nanoscale tip attached to a flexible cantilever scans the sample surface, and atomic interactions between the tip and the surface are measured to reconstruct the geometry of the sample. The resulting image intensity corresponds to variations in surface height. Nanofluidic devices are optical systems incorporating miniature fluidic circuits that enable the observation and manipulation of single molecules and nanoscale particles, often exploiting electrokinetic mechanisms to highlight nanostructures within the sample. Images acquired from these devices are typically grayscale; in some cases, they are recolored post-acquisition to enhance visualization and facilitate interpretation of distinct DNA structures.

Despite the high resolution of modern imaging systems, DNA inspection requires careful sample preparation, including staining, labeling, or embedding in specific solutions to optimize visualization (Kang et al., 2025).

Over the past decade, biomedical image analysis has greatly benefited from deep learning methods (Xing et al., 2017; Min et al., 2017; Ramsundar et al., 2019). These approaches have enabled tasks such as image enhancement, reconstruction, classification, and object or structure detection. Deep learning techniques are particularly well suited for microscopy data, which are characterized by high dimensionality, complex structural patterns, and intertwined chemical and temporal relationships (Xing et al., 2017).

In the context of DNA research, deep learning has been applied to tasks including DNA motif mining (He et al., 2021), DNA sequencing and analysis (Abd-Alhalem et al., 2021; Eraslan et al., 2019; Ramu et al., 2024), sequence registration (Vinodhini et al., 2018), and DNA damage detection and classification (Kang et al., 2025; Ergen and Ceyani, 2018). Several deep learning approaches have been proposed to classify microscopy images of DNA samples as damaged or intact and, when damage is present, to assess its severity. Convolutional neural networks (CNNs) are particularly effective because convolutional operations enable multi-scale analysis of structural features, which is essential for assessing DNA integrity. In typical workflows, a CNN receives an image as input and outputs a label indicating the presence of damage and, if applicable, its characteristics (e.g., morphological features and degree of damage). Such networks consist of multiple layers implementing tensor operations (convolutions) combined with nonlinear activation functions and interleaved pooling layers that reduce redundancy and dimensionality. The parameters of these linear and nonlinear operators are *learned* by training the network on a large annotated dataset (the *training set*) and minimizing a loss function that measures the discrepancy between predicted outputs and ground-truth annotations. After this *training phase*, the network can classify new, previously unseen images according to the presence and type of DNA damage.

The *training phase* is particularly delicate and requires careful control of several variables. First, the training dataset must contain a sufficient number of representative examples. Too few examples may fail to capture the variability of regions exhibiting DNA damage, whereas excessively large or redundant datasets may increase the risk of overfitting and reduce the model’s ability to generalize to unseen damage types. Moreover, the dataset should be balanced, ensuring that all damage classes are adequately represented. Images must also be carefully selected to avoid ambiguous annotations that may confuse the model during learning. Second, DNA damage regions may appear under highly variable conditions. Samples can differ in scale, orientation, staining intensity, and imaging quality, and images may be affected by noise arising from imperfect sample preparation or suboptimal microscope settings. To improve generalization, data augmentation techniques are commonly applied. These include geometric transformations (e.g., rotation, scaling, translation), intensity modifications, and controlled noise injection. Augmentation can also be performed using deep learning–based generative approaches. Generative adversarial networks (GANs), variational autoencoders (VAEs), and transfer learning strategies—including domain transfer and domain adaptation—have been proposed to enhance training datasets and improve model robustness (Pavan Kumar and Jayagopal, 2021; Jabbar et al., 2021; Zhang and Gao, 2022; Akkem et al., 2024; HassanPour Zonoozi and Seydi, 2023; Bengesi et al., 2024). Third, accurate data annotation is critical. Annotation errors must be minimized to ensure reliable model training and detection performance. Although annotation is primarily performed by expert biologists, software tools are available to assist in this process (Tiano et al., 2005), including systems that incorporate transfer learning and domain adaptation techniques to improve labeling consistency.

### 5.1 DNA damage detection from *comet assay* images

**FIGURE 7:**
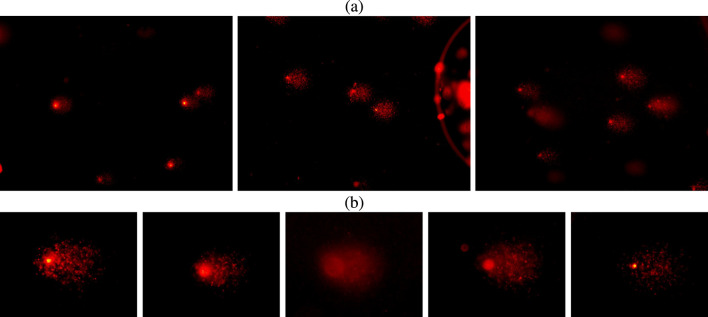
Representative *comet assay* images and DNA comet variability. **(a)** Examples of *comet assay* images from the repository in (Yiyi, 2025). The images contain multiple DNA comets and, in some cases, noise due to imperfections in the staining procedure or improper imaging settings (see the image in the middle). **(b)** Examples of individual DNA comets cropped from *comet assay* images in (Yiyi, 2025). The comets exhibit substantial variability in intensity, granularity, and shape.

**FIGURE 8:**
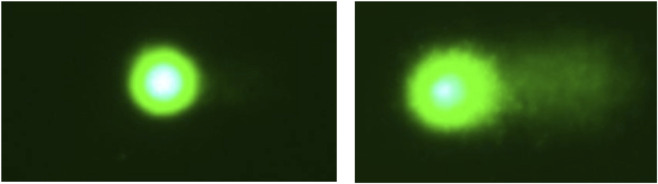
Example of *comet assay* images from the repository in (Hájek, 2025). The sample on the left does not present detectable DNA damage, as it appears as a compact, spherical head without a visible tail. In contrast, the comet-shaped sample on the right indicates the presence of DNA damage.

The *comet assay* (Cordelli et al., 2021), also known as single-cell gel electrophoresis, is a widely used technique for detecting and quantifying DNA strand breaks at the level of individual cells. In this assay, cells embedded in agarose are lysed to remove membranes and proteins, and the remaining nucleotides are subjected to electrophoresis. Under these conditions, fragmented or relaxed DNA migrates toward the anode, forming a characteristic comet-like structure composed of a head and a tail (Olive and Banáth, 2006; Collins, 2004). The morphology of the comet provides both qualitative and quantitative information on DNA damage severity. As DNA damage increases, the tail becomes progressively longer and more intense, while the head decreases in size and fluorescence intensity (see Figures 7, 8). Tail length, tail intensity, and their product—commonly referred to as the tail moment—are widely used indicators of DNA damage (Kumaravel and Jha, 2006; Kumaravel et al., 2009; Collins et al., 2023). In cases of severe DNA damage, the tail may dominate the image entirely and the head may become barely visible or disappear, a pattern typically associated with extensive strand breakage or cytotoxic effects (Olive et al., 1990; Collins, 2004).

Based on these visual features, *comet assay* images are commonly categorized using semi-quantitative scoring systems. A widely adopted approach defines five damage classes, ranging from class 0 (no detectable DNA damage) to class 4 (severe DNA fragmentation), corresponding to increasing tail length and DNA content in the tail (Collins et al., 1995; Collins, 2004). In addition to visual scoring, quantitative descriptors such as tail length, percentage of DNA in the tail, and tail moment provide continuous measures of DNA damage and are routinely used in both manual and automated analyses (Olive and Banáth, 2006).

AI and machine learning techniques have been increasingly applied to automate *comet assay* image analysis by detecting individual comets and extracting morphological and intensity-based features associated with DNA damage. These tools are primarily designed to identify comets in microscopy images and classify them as representing intact or damaged DNA based on head–tail geometry, fluorescence distribution, and texture features. In some cases, regression or multi-class classification models are also employed to estimate damage severity or assign comets to discrete damage categories consistent with established scoring schemes (Collins, 2004; Olive and Banáth, 2006). By leveraging these well-defined morphological correlates of DNA damage, AI-based approaches improve throughput, reproducibility, and objectivity in *comet assay* analysis.

Several deep learning–based tools for comet detection and DNA damage classification have been published in recent years, including DeepComet (Hong et al., 2020), a Faster R-CNN–based approach (Rosati et al., 2020), GamaComet (Anarossi et al., 2022), Comet Analyser (Beleon et al., 2022), and an ensemble deep learning method described in (Mehta et al., 2023). All these software tools—whose main characteristics are summarized in Table 3 - segment comets from input images using neural network architectures based on ResNet (He et al., 2016) (from *Res*idual *Net*work) and classify the extracted regions according to comet-specific features. ResNet architectures are widely used in medical imaging to support diagnostic tasks (Xu et al., 2023). ResNet was originally developed to address the degradation in accuracy observed in very deep neural networks, a problem not primarily caused by overfitting but by numerical instabilities associated with vanishing gradients during training. To overcome this limitation, ResNet introduced shortcut connections into the network architecture. These connections are parameter-free computational paths that mitigate the vanishing gradient problem by facilitating gradient propagation across layers. Rather than learning a direct mapping h(x) from input x to output, ResNet learns a *residual function* R(x)=h(x)−x , such that the output of a residual block is given by h(x)=R(x)+x . This formulation prevents gradients from becoming excessively small as they propagate through deep network layers, thereby enabling effective training of very deep architectures. ResNet architectures are commonly denoted as ResNet-X, where X indicates the number of layers used in the network.

**TABLE 3:** Summary of the five deep learning models for comet segmentation and classification described in Section 5.1.

DL tool	DL model	Output	Tested on	Code availability
DeepComet (Hong et al., 2020)	Mask R-CNN (Abdulla, 2017) with ResNet-50 as backbone	Binary classification in Ghost and Non-Ghost Cells	Canine peripheral blood mononuclear cells of six beagle	NA
Faster R-CNN (Rosati et al., 2020)	Faster R-CNN (Ren et al., 2016) with ResNet-101	Classification of the DNA damage level as low, medium or high	Bovine lymphocytes exposed to γ radiations and neonatal foreskin human dermal fibroblasts exposed to UV radiation	Rosati (2025)
GamaComet (Anarossi et al., 2022)	Faster R-CNN (Ren et al., 2016) with ResNet-50	Classification of DNA damages in 5 levels	Cells from Buccal Mucosa after Dental Radiography	Anarossi et al. (2025)
Comet Analyzer (Beleon et al., 2022)	Manual,Semi-Automatic or Automatic comet segmentation with ResNet-50; Manual or Automatic comet classification via hand-crafted or machine learning methods	Arbitrary levels of DNA damages as input by the user	Cancer Cells	Beleon et al. (2025)
Ensemble Model (Mehta et al., 2023)	Ad-hoc CNN + VGG-19 (Simonyan and Zisserman, 2014) + Xception (Chollet, 2017)	Amount of the DNA damage s	Human pluripotent stem cells	NA

DeepComet (Hong et al., 2020) performs binary classification of DNA damage using the Mask R-CNN architecture (Abdulla, 2017). Mask R-CNN segments the input image into regions of interest via a Region Proposal Network (RPN), which employs ResNet-50 (Liang, 2020) as a backbone to extract multiscale features and generate bounding boxes likely to contain comets. Each detected region is classified as a ghost or non-ghost comet. Ghost cells correspond to comets with a distinct head and tail, indicative of severe DNA damage, whereas non-ghost cells exhibit negligible or absent heads and broad tails, corresponding to intact DNA. For ghost cells, DeepComet further segments the head and tail and computes quantitative measures such as the percentage of DNA in the tail, tail moment, and Olive moment (Olive et al., 1990). The model was evaluated on 1,037 images containing 8,271 comets from canine peripheral blood mononuclear cells and demonstrated robustness to noise caused by agarose gel preparation artifacts (Hong et al., 2020).

The Faster R-CNN–based approach in (Rosati et al., 2020) adapts the object detection framework of (Ren et al., 2016) to classify three levels of DNA damage (low, medium, and high) from 529 images containing multiple comets. Experiments were conducted on two datasets: bovine lymphocytes exposed to γ -radiation and neonatal foreskin human dermal fibroblasts exposed to UV radiation. Annotations were generated using the Marche software (Tiano et al., 2005) and refined by three expert biologists. While the method outperformed several state-of-the-art approaches on average, performance varied across damage classes and datasets, highlighting the importance of balanced and representative training data.

GamaComet (Anarossi et al., 2022) is a freely available tool implementing Faster R-CNN with ResNet-50 for comet detection and classification. It was evaluated on 279 images from buccal swab samples of 24 patients, containing 519 comets across five damage levels. Due to class imbalance and limited data availability, geometric augmentation and transfer learning were employed to improve performance.

Comet Analyser (Beleon et al., 2022) provides manual, semi-automatic, and automatic comet segmentation and classification. In automatic mode, segmentation is performed using a ResNet-50 model pretrained on 5,000 annotated cancer cell images, although users may retrain the network on custom datasets. Classification can be performed manually or using decision trees, k-nearest neighbors, naive Bayes, or support vector machines. The tool computes 21 quantitative features for the head, tail, and entire comet, including area, elongation, intensity statistics, sphericity, Olive moment, and DNA percentage. Notably, Comet Analyser allows an arbitrary number of damage classes, rather than being restricted to the five categories proposed in (Collins et al., 1995).

Finally, the ensemble method proposed in (Mehta et al., 2023) combines a custom convolutional neural network with VGG-19 (Simonyan and Zisserman, 2014) and Xception (Chollet, 2017). Ensemble modeling aggregates predictions from multiple architectures to reduce error and improve robustness. The model outputs a continuous DNA damage score derived from 14 visual features, including length, area, intensity, and DNA percentage of the head, tail, and entire comet. Trained on 1,047 augmented images and tested on 131 images of human pluripotent stem cells, the ensemble approach demonstrated superior performance compared with single-model solutions.

### 5.2 DNA damage detection from images of ionizing radiation-induced (repair) foci (IRIF)

**FIGURE 9:**
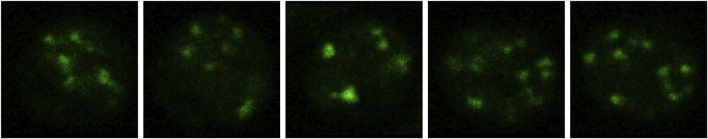
Examples of IRIF images from the repository in Wanotayan et al. (2022c). From left to right, the radiation dose increases from 1 Gy to 5 Gy.

An alternative approach to detecting DNA damage is to monitor DNA repair processes directly. Exposure to ionizing radiation or radiomimetic chemicals can induce severe DNA lesions, including double-strand breaks (DSBs), which are potentially lethal to cells. In response, cells activate DNA damage response pathways and recruit repair proteins to the sites of damage. These proteins form localized nuclear regions, known as *nucleos foci*, around damaged DNA sites (Horn and Rothkamm, 2011). Such foci—observable using standard confocal fluorescence microscopy—serve as sensitive markers of DNA damage. Quantifying their number and analyzing their properties, such as size, shape, and intensity, provides information on the presence, severity, and temporal evolution of DNA lesions. Ionizing radiation–induced (repair) foci (IRIFs) represent one of the most sensitive assays for detecting DSBs (see Figure 9). These foci arise following exposure to ionizing radiation and are composed of DNA repair proteins such as γ H2AX (phosphorylated histone H2AX) (Rothkamm and Horn, 2009) and 53BP1 (tumor suppressor p53-binding protein 1) (Wang et al., 2002), which are recruited to damaged chromatin in a tightly regulated spatial and temporal manner.

As with *comet assay* analysis, manual inspection of IRIF images is time-consuming and prone to observer bias and variability. Consequently, deep learning–based approaches for IRIF image analysis have emerged as effective alternatives. In this context, the analysis typically involves segmenting foci from confocal microscopy images, counting the number of foci per nucleus, and extracting additional features such as brightness, size, shape, and boundary smoothness. The temporal evolution of these features provides insight into DNA repair progression and efficiency.

Several deep learning–based tools have been proposed for IRIF detection and analysis, including CellProfiler 3.0 (McQuin et al., 2018), FociNet (Chen et al., 2020a), DeepFoci (Vicar et al., 2021b), and FociRad (Wanotayan et al., 2022a). A comparative summary of these methods is provided in Table 4.

**TABLE 4:** Summary of the four deep learning models for foci detection and DNA damage evaluation described in Section 5.2.

DL tool	DL model	Output	Tested on	Code availability
CellProfiler 3.0 (McQuin et al., 2018)	UNet and MeasureImageFocus	Foci Number and Morphology	NA	McQuin et al. (2020)
FociNet (Chen et al., 2020a)	UNet for foci segmentation; VGG-19 for foci classification	Cell classification as normal, damaged and non-signaling; Foci Number, IR dose estimate	heLa = EGFP-53BP1 cells	Chen et al. (2020b)
DeepFoci (Vicar et al., 2021b)	3D-UNet + MSER (Parvati et al., 2008)	Foci number, location and other features	head and neck tumor cells; mesenchymal NHDFs and radioresistant U-87	Vicar et al. (2021a)
FociRad (Wanotayan et al., 2022a)	YOLO-v4	Foci number and radiation dose estimation	X-ray Irradiated blood cells	Wanotayan et al. (2022b)

CellProfiler 3.0 (McQuin et al., 2018) is an advanced version of the open-source CellProfiler software originally introduced in 2006 (Carpenter et al., 2006). It extends earlier functionality by supporting robust 3D image processing and integrating machine learning and deep learning modules. Of particular relevance to IRIF detection is the ClassifyPixels-Unet plugin, which implements a UNet-based segmentation algorithm to classify image pixels into background, nucleus interior, and nucleus boundary classes. UNet is a widely adopted architecture in medical imaging due to its ability to extract contextual and semantic features efficiently, its modular and symmetric design, and its effectiveness with relatively small annotated datasets (Azad et al., 2024). Originally proposed by Ronneberger et al., in 2015, UNet consists of an encoder–decoder architecture in which contextual information is captured through successive convolution and pooling operations and then combined with high-resolution features during decoding. Extensions of UNet to volumetric data, such as 3D-UNet (Çiçek et al., 2016), are particularly relevant for biological and medical imaging applications.

In addition to UNet-based segmentation, CellProfiler 3.0 includes the MeasureImageFocus module, a trainable deep learning–based component developed in collaboration with Google Accelerated Science for foci detection. The software has been validated on several case studies, including 3D image stacks of DNA-stained nuclei from human induced pluripotent stem cells, mouse embryo blastocysts, and mouse trophoblast stem cells (McQuin et al., 2018). The source code is publicly available (McQuin et al., 2020).

FociNet (Chen et al., 2020a) focuses on detecting and classifying foci formed by EGFP-tagged 53BP1. Fluorescence microscopy images are first rescaled to standardize resolution, and contrast enhancement is applied to improve signal detection. Cell segmentation is performed using UNet, after which the VGG-19 architecture is applied to classify each nucleus as normal, damaged, or non-signaling. Unlike approaches trained on full-field images, FociNet trains and validates the classifier on manually annotated single-nucleus images, reducing bias introduced by global image context. The model was trained and tested on HeLa-EGFP-53BP1 cells exposed to ionizing radiation and demonstrated the ability to quantify damage levels and assess radioprotective effects of compounds such as WR-1065 dihydrochloride (WR-1065 HCl) (Chen et al., 2020a).

DeepFoci (Vicar et al., 2021b) employs a 3D-UNet architecture to extract IRIFs directly from three-dimensional image stacks rather than from two-dimensional projections. Each stack includes channels for nuclear staining and IRIF markers ( γ H2AX and 53BP1). Nuclear regions and foci are segmented using separate UNet models, with additional morphological processing and maximally stable extremal region (MSER) detection used to isolate individual foci (Parvati et al., 2008). The method also computes quantitative descriptors such as foci number, intensity, volume, solidity, and circularity. DeepFoci was validated on multiple cell types, including head and neck cancer tissues, mesenchymal fibroblasts, and radioresistant glioblastoma cells, with datasets and code made publicly available (Vicar et al., 2021a; Vicar et al., 2021b; Vicar et al., 2021c).

FociRad (Wanotayan et al., 2022a) applies the You Only Look Once version 4 (YOLO-v4 object detection framework (Bochkovskiy et al., 2020)) to detect γ H2AX foci. YOLO is a single-stage detector that predicts object locations and classes in a single pass, enabling high computational efficiency (Redmon et al., 2016). In FociRad, YOLO-v4 is trained to (i) identify individual nuclei in full-field images and (ii) detect foci within cropped nuclei. DNA damage is quantified by counting detected foci and estimating radiation dose. The dataset and implementation are publicly available (Wanotayan et al., 2022a; Wanotayan et al., 2022b).

While these methods provide objective and reproducible quantitative measures of DNA damage, biological interpretation remains essential for understanding DNA repair efficiency, pathway engagement, and downstream cellular outcomes. Beyond automated detection.

AI and machine learning approaches enable systematic quantification of morphological, spatial, and temporal features that are directly relevant to DNA repair biology. Commonly extracted descriptors include foci number, size, intensity, shape, spatial clustering, and persistence over time, all of which serve as biologically meaningful proxies for damage complexity and repair pathway activity (Rogakou et al., 1998; Bonner et al., 2008).Small, discrete, and uniformly distributed γ H2AX or 53BP1 foci are typically associated with isolated DSBs that are efficiently repaired, often via canonical non-homologous end joining. In contrast, large, dense, or irregularly shaped foci generally reflect clustered or complex DNA damage that is more challenging to repair and may require homologous recombination or prolonged damage signaling (Löbrich and Jeggo, 2007). Radiation quality also influences foci morphology and repair dynamics: low–linear energy transfer radiation induces sparse, punctate foci, whereas high-LET radiation produces dense, track-like foci associated with delayed repair and increased cell lethality (Asaithamby and Chen, 2009; Nikitaki et al., 2016). Finally, the persistence of foci 24–48 h after damage induction is widely recognized as a marker of irreparable lesions and is predictive of senescence, apoptosis, or mitotic catastrophe. By enabling longitudinal analysis of foci dynamics, AI-based approaches facilitate the linkage of early damage signatures to long-term cellular fate decisions (Bonner et al., 2008; Löbrich and Jeggo, 2007).

### 5.3 Final remarks and perspective on DNA damage detection from images

Our analysis of the current state of the art in software tools for detecting and classifying DNA damage from images highlights several key challenges that deep learning–based methods must address carefully.

1. *Data scarcity*: Only a limited number of datasets containing comet or foci images are currently available, and access to these datasets is often constrained by privacy and anonymization policies. This scarcity, together with the relatively small number of images per dataset, necessitates the use of data augmentation techniques. Common augmentation strategies generate rescaled, translated, rotated, or flipped versions of existing images. While these transformations improve robustness to geometric variations, they do not create fundamentally new instances representing different degrees or types of DNA damage. To partially address this limitation, some approaches artificially modulate damage severity by altering the intensity of comet regions or foci. For example (Namuduri et al., 2019) proposes a hand-crafted method to generate synthetic comet images by combining geometric primitives (circles and triangles) with tunable size, aspect ratio, and intensity to simulate varying damage levels. By contrast, to the best of our knowledge, no comparable methods have been developed to artificially generate foci images or systematically alter foci number and spatial organization. Generative-model–based augmentation can improve robustness in segmentation and detection tasks, but it carries the risk of producing synthetic samples that do not correspond to biologically realistic scenarios. In addition, domain adaptation and transfer learning remain essential to accelerate training and improve generalization across datasets.
2. *Quality of available data and annotation*: Available datasets exhibit substantial heterogeneity in both image quality and annotation strategies. As discussed in Section 5.1, some comet-analysis methods provide binary classifications (e.g., intact versus damaged DNA (Hong et al., 2020)), whereas others output continuous damage scores (e.g., values between 0 and 1 in (Mehta et al., 2023)). Furthermore, *comet assay* protocols are not fully standardized (for example, alkaline versus neutral *comet assays*), and different imaging platforms are used, resulting in variability in image resolution, contrast, and noise characteristics. Similar issues arise in IRIF-based approaches, where different biomarkers (e.g., γ H2AX, 53BP1, or both) and distinct quantitative features are employed to assess damage severity. In both comet and foci imaging, noise introduced by imperfect sample preparation or suboptimal microscope settings remains a critical challenge. Consequently, effective image denoising is often a necessary preprocessing step prior to segmentation and analysis.
3. *Comparison of methods*: Data scarcity, coupled with heterogeneity in image quality and annotation schemes, makes it difficult to perform fair and systematic comparisons among deep learning models proposed in the literature. In many studies, methods are trained and evaluated on highly specific case studies, limiting the generalizability of reported performance metrics. This issue is further compounded when source code or trained models are not made publicly available, hindering reproducibility and independent validation.
4. *Classification Independent of Causative Agents* In order to identify lesion types associated with specific damaging agents, enzyme-modified comet assays, i.e., standard assays combined with enzymes like e.g., Fpg and EndoIII, have been introduced (Muruzabal et al., 2021). Nevertheless, no deep learning frameworks have yet been developed to analyze images derived from such modified assays. Extending current models to distinguish damage mechanisms or causative agents—rather than merely classifying damage severity—represents an important and largely unexplored research direction.

Finally, it is worth noting that, while IRIF detection traditionally relies on monitoring DNA repair proteins, recent evidence suggests that biomolecular condensates may serve as alternative or complementary biomarkers of DNA damage (Fijen and Rothenberg, 2021). These condensates, which form under conditions of oxidative stress and participate in enzymatic reactions, appear as phase-separated structures within DNA damage foci. Their dynamic behavior and sensitivity to cellular context make them promising targets for future image-based studies of DNA damage and repair mechanisms, particularly when combined with advanced deep learning and uncertainty-aware modeling approaches.

## 6 Conclusion

Artificial intelligence (AI) techniques and methodologies show considerable promise for automating the analysis and prediction of data related to DNA damage and repair mechanisms. These approaches can be applied across several key areas: (i) predictive modeling, to forecast how cells repair DNA lesions as a function of damage type, cellular context, and genetic background; (ii) image analysis, where AI streamlines the interpretation of microscopy data—such as repair foci formation—to quantify DNA damage and enable high-throughput screening of compounds that influence repair processes; (iii) data integration and analysis, combining large datasets from multiple assays to uncover patterns of DNA damage induced by environmental and chemical factors, thereby elucidating chemico-biological interactions; (iv) genetic analysis, to identify genetic determinants that modulate DNA repair efficiency and disease susceptibility; and (v) computational simulation, in which AI complements molecular dynamics and mechanistic modeling to study the behavior of DNA repair enzymes and predict the functional impact of genetic variants. Despite these advances, substantial challenges remain. AI models often struggle to fully capture the complex, context-dependent, and stochastic nature of biological systems. Limitations persist in modeling rare or heterogeneous damage types and in extrapolating from restricted or biased datasets. Over-simplification of DNA repair pathways may lead to misinterpretation of results, while the reliance on large, high-quality datasets constrains the ability of models to generalize to novel or complex damage scenarios. Future research should therefore move beyond the sole automation of analytical pipelines and focus on developing AI frameworks that more effectively integrate biological complexity, explicitly handle uncertainty and stochasticity, and balance data-driven prediction with mechanistic insight. Such advances—summarized and discussed in Table 5—will be essential for making AI-based tools more robust, generalizable, and biologically meaningful in the study of DNA damage and repair.

**TABLE 5:** Summary of the key challenges in the application of AI to DNA damage research together with representative near-term technical approaches that may help address them.

Challenge	Description	Potential near-term technical approaches
Uncertainty modeling as a central requirement	Experimental data and computational models used to investigate DNA repair mechanisms and evaluate DNA damage are inherently affected by biological variability, stochasticity, and measurement uncertainty. Explicitly modelling and quantifying these sources of uncertainty is essential for reliable predictions in biologically and clinically meaningful contexts	Bayesian neural networks (and its approximation as Monet Carlo dropout (Gal and Ghahramani, 2016)), probabilistic graphical models, predictive uncertainty calibration techniques (e.g., quantile regression (Xu, 2023)) and uncertainty-aware ensemble models (Vrugt and Robinson, 2007)
Sequential modeling of damage and repair dynamics	DNA repair processes evolve over time and involve sequential molecular events such as signaling activation, protein recruitment, and lesion resolution. Capturing these temporal dependencies is essential for understanding repair efficiency and predicting cellular outcomes following genotoxic stress	Recurrent neural networks (RNNs) and bidirectional RNNs (BRNNs), time-series modeling frameworks, attention-based sequence models (e.g., (Shen et al., 2018)), and integration of longitudinal imaging or omics datasets
Beyond detection toward mechanism-aware classification	Most AI-based approaches focus on detecting and quantifying DNA damage based on visual features, without identifying the causative agents or underlying repair pathways. Linking observed patterns to biological mechanisms remains a key challenge	Hybrid models combining deep learning with pathway knowledge (e.g., Bayesian networks or agent-based models), multi-modal learning integrating molecular and imaging data, and enzyme-modified *comet assay* (Muruzabal et al., 2021) analysis with AI-based classification
Data quality, annotation, and benchmarking	Reliable identification and assessment of DNA damage depend on the availability of high-quality datasets, including well-acquired images and accurate annotations. Poor image quality or inconsistent labeling may lead to misdetection and misclassification in comet assay or foci analysis	Development of community benchmark datasets, standardized annotation protocols, shared repositories for *comet assay* and IRIF imaging data (e.g., (Liang et al., 2024)), and collaborative data curation initiatives similar to benchmark datasets used in computer vision
